# Дефицит витамина D в России: первые результаты регистрового неинтервенционного исследования частоты дефицита и недостаточности витамина D в различных географических регионах страны

**DOI:** 10.14341/probl12736

**Published:** 2021-04-05

**Authors:** Л. А. Суплотова, В. А. Авдеева, Е. А. Пигарова, Л. Я. Рожинская, Е. А. Трошина

**Affiliations:** Тюменский государственный медицинский университет; Тюменский государственный медицинский университет; Национальный медицинский исследовательский центр эндокринологии; Национальный медицинский исследовательский центр эндокринологии; Национальный медицинский исследовательский центр эндокринологии

**Keywords:** витамин D, распространенность, эпидемиология

## Abstract

ОБОСНОВАНИЕ. В Российской Федерации отсутствуют широкомасштабные одномоментные многоцентровые эпидемиологические исследования, оценивающие распространенность дефицита и недостаточности витамина D в различных географических широтах. Недостаточный уровень солнечной инсоляции и неадекватное содержание витамина D в продуктах питания диктуют необходимость изучения эпидемиологической структуры низкого статуса витамина D в России.ЦЕЛЬ. Оценить частоту дефицита и недостаточности витамина D среди населения, проживающего в регионах Российской Федерации, расположенных в широтах от 45° до 70°.МАТЕРИАЛЫ И МЕТОДЫ. Проведен первый этап российского многоцентрового неинтервенционного регистрового исследования по методу «поперечных срезов» с марта 2020 по май 2020 гг.РЕЗУЛЬТАТЫ. В регионах РФ, которые представляют собой репрезентативную с географической точки зрения выборку с высоким риском развития низкого уровня витамина D, его дефицит отмечен в 55,96%, а уровень дефицита и недостаточности регистрируется в 84,01%.ЗАКЛЮЧЕНИЕ. Пристальное внимание к широкому масштабу проблемы дефицита и недостаточности витамина D в РФ будет способствовать прогрессивному формированию различных образовательных и профилактических программ, необходимых для укрепления здоровья и улучшения качества жизни населения.

## Abstract (en)

BACKGROUND. In Russian Federation, there are no large-scale cross-sectional multicenter epidemiological studies assessing the prevalence of vitamin D deficiency and insufficiency in different geographical latitudes. Insufficient solar insolation and inadequate vitamin D content in food dictate the need to study the epidemiological structure of low vitamin D status in Russia.AIM. To assess the incidence of vitamin D deficiency and insufficiency among the population living in the regions of the Russian Federation located at latitudes from 45 ° to 70 °.MATERIALS AND METHODS. The first stage of the Russian multicenter non-interventional registry study using the «crosssectional» method was carried out from March 2020 to May 2020.RESULTS. In regions that represent a geographically representative sample of regions of the Russian Federation with a high risk of developing low levels of vitamin D, it’s deficiency was noted in 55.96%, and the level of deficiency and insufficiency was recorded in 84.01%.CONCLUSION. Close attention to the wide scale of the problem of vitamin D deficiency and insufficiency in the Russian Federation will contribute to the progressive formation of various educational and preventive programs necessary to strengthen health and improve the quality of life of the population.

## ОБОСНОВАНИЕ

В процессе обогащения фундаментальных научных данных о значении дефицита витамина D для здоровья человека расширялись горизонты его клинического применения в практической медицине. История исследований витамина D началась в преддверии XX в. и продолжается до сих пор. В настоящее время витамин D насчитывает фантастическую траекторию столетнего пути от «витамина для роста костей», применяемого исключительно в детской практике, до главной цели крупномасштабных комплексных полногеномных исследований, которая вносит значительный вклад в профилактику и лечение обширного круга социально значимых заболеваний [1–6]. Однако, несмотря на свою вековую исследовательскую историю, накопленный клинический опыт применения витамина D в разных областях медицинской науки и практическом здравоохранении, а также неотъемлемый вклад в состояние здоровья населения, во всем мире колоссально острой остается проблема широкой распространенности дефицита витамина D [7]. Климатогеографические особенности региона проживания играют ключевую роль в развитии дефицита и недостаточности витамина D у местного населения. Объясняется это тем, что дефицит витамина D широко распространен на территориях, располагающихся в северных широтах (выше 35° с.ш.), где из-за низких среднегодовых температур, небольшого количества солнечных дней, а также острого угла падения солнечных лучей и их рассеивания в верхних слоях атмосферы в осенне-зимний и ранний весенний период соприкосновение с кожными покровами проходит по касательной, что значительно снижает возможность адекватной выработки витамина D [8]. Мировая практика многоцентровых популяционных исследований подтверждает высокую частоту пандемически низкого статуса витамина D среди всех возрастных групп, эти сведения находят отражение и в результатах российских исследований, проведенных с прицелом на различные географические широты [9–18]. Вместе с тем для полномасштабной оценки и объемной эпидемиологической характеристики распространенности дефицита и недостаточности витамина D на территории Российской Федерации (РФ) имеющихся данных недостаточно. К тому же их совокупный анализ весьма затруднен в связи с отсутствием единой стратегической концепции опубликованных исследований.

## ЦЕЛЬ ИССЛЕДОВАНИЯ

Цель настоящего исследования — оценить частоту дефицита и недостаточности витамина D среди населения, проживающего в регионах Российской Федерации, расположенных в широтах от 45° до 70°.

## МАТЕРИАЛЫ И МЕТОДЫ

Место и время проведения исследования

Место проведения. РФ. Регионы, расположенные в широте от 45° до 70° (г. Москва, Ростов-на-Дону, Санкт-Петербург, Мурманск, Екатеринбург, Тюмень, Кызыл, Владивосток, Норильск, Новосибирск).

Время исследования. Первый этап исследования включал весенний период с 01 марта 2020 по 31 мая 2020.

Изучаемые популяции

Популяция. Здоровые добровольцы (добровольцы, сообщившие при даче информированного согласия об отсутствии сопутствующих заболеваний в стадии обострения, тяжелых, декомпенсированных или нестабильных соматических заболеваний, острых воспалительных заболеваний или хронических воспалительных заболеваний в стадии обострения на момент скрининга, инвалидности, операций на органах ЖКТ).

Критерии включения.

1. Добровольцы мужского и женского пола в возрасте от 18 до 50 лет включительно.

2. Индекс массы тела (ИМТ) в пределах 18,5–30,0 кг/м2 включительно при массе тела свыше 45 кг и не более 100 кг включительно.

3. Наличие подписанной Формы информированного согласия на участие в исследовании.

Критерии исключения.

1. Доброволец в настоящее время участвует в каком-либо другом клиническом исследовании.

2. Прием добровольцем лекарственных средств или биологически активных добавок, содержащих витамин D в форме монопрепаратов или комбинаций витамина D с кальцием.

3. Клинические признаки синдрома мальабсорбции на момент скрининга (например, диарея, боли в животе, астеновегетативный синдром и т.д.).

4. Беременность или период грудного вскармливания.

5. Неспособность добровольца, по мнению сотрудника исследовательского центра, выполнить условия данного исследования.

6. Прочие условия, которые, по мнению сотрудника исследовательского центра, препятствуют включению добровольца в исследование.

Способ формирования выборки из изучаемой популяции

Набор добровольцев проводился в центрах ООО «ИНВИТРО», компаний, оказывающих медицинские услуги населению под товарными знаками ИНВИТРО® и INVITRO® на основании лицензионных договоров, сублицензионных договоров/договоров коммерческой концессии или привлеченных ООО «ИНВИТРО» к исследованию, расположенных в различных регионах страны. Участников включали последовательно на протяжении ограниченного периода времени. Исследование проведено по единому протоколу во всех исследовательских центрах.

Дизайн исследования

Российское многоцентровое неинтервенционное регистровое исследование.

Первичная конечная точка: уровень 25(ОН)D в сыворотке крови.

Вторичная конечная точка: демографическая характеристика участников исследования (возраст, пол, раса).

Описание медицинского вмешательства

В рамках данного исследования медицинское вмешательство пациентам не проводилось.

Методы

Методика определения уровня 25(ОН)D в сыворотке крови. Анализ крови на 25(ОН)D производился методом хемилюминесцентного иммуноанализа на микрочастицах в центрах ООО «ИНВИТРО». Согласно интерпретации Российской ассоциации эндокринологов 2015 г., уровень 25(ОН)D расценивался как адекватный при показателе ≥30 нг/мл (≥75 нмоль/л), недостаточность — ≥20 и <30 нг/мл (≥50 и <75 нмоль/л), дефицит — <20 нг/мл (<50 нмоль/л), выраженный дефицит витамина D — <10 нг/мл (<25 нмоль/л). Референсный интервал определения: 3,4–155,9 нг/мл.

Статистический анализ

Размер выборки вычислялся с целью обеспечения достаточной точности при оценке качественных критериев. Анализ стратифицирован по регионам. В зависимости от региона включались от 42 до 47 участников. Статистический анализ проводился с помощью специализированного программного обеспечения StatSoft STATISTICA и включал оценку следующих параметров: анализ лабораторных данных и демографических показателей. Описательная статистика количественных признаков представлена медианами (в формате Me). При сравнении двух независимых групп по количественному признаку для оценки статистической значимости межгрупповых различий использован U-тест Манна–Уитни (U). Связь количественных переменных оценивалась с помощью коэффициента корреляции Спирмена. Для сравнения групп по качественному признаку использован расчет 95% доверительного интервала для отношения шансов и тест χ² Пирсона. Для статистического «взвешивания» использовалась актуальная информация о численности населения в зависимости от возраста и региона, представленная на сайте Федеральной службы государственной статистики («Численность населения Российской Федерации по полу и возрасту», url: https://www.gks.ru/compendium/document/13284 [дата обращения: 09.07.2020]). Критический уровень значимости при проверке статистических гипотез принимался равным 0,05.

Этическая экспертиза

Настоящее исследование проведено строго в соответствии с этическими принципами, провозглашенными в Хельсинкской декларации, ICH GCP (МКГ ККП) и действующим законодательством РФ. Протокол исследования № AQ-01/20, версия 2.0 от 25 февраля 2020 г. был одобрен Независимым междисциплинарным Комитетом по этической экспертизе клинических исследований.

## РЕЗУЛЬТАТЫ

Объекты (участники) исследования

В промежуточный анализ результатов 1-го этапа исследования (весна 2020 г.) вошли данные 445 субъектов от 18 до 50 лет из 10 регионов РФ. Наибольшее число добровольцев было набрано в Санкт-Петербурге и Тюмени (по 47 человек), наименьшее — в Екатеринбурге и Ростове-на-Дону (по 42 человека). Исходные характеристики участников исследования по регионам представлены в таблице 1.

**Table:** Таблица 1. Распределение субъектов исследования по географическим регионам

Географический регион	Мужчины	Женщины	Всего
Владивосток	12	32	44
Екатеринбург	11	31	42
Западное Заполярье	10	36	46
Кызыл	14	31	45
Москва	8	37	45
Новосибирск	6	38	44
Норильск	13	30	43
Ростов-на-Дону	8	34	42
Санкт-Петербург	8	39	47
Тюмень	15	32	47
Всего в исследовании	105	340	445

Основные результаты исследования

Распространенность дефицита и недостаточности витамина D в исследуемых регионах. Согласно результатам проведенного исследования, уровень недостаточности 25(OH)D был зарегистрирован у 27,87% добровольцев, дефицит — у 56,40%. Суммарно у 84,27% обследованных установлен показатель низкого статуса витамина D, а оптимальный уровень диагностирован у 15,73%. В целом по исследованию средний уровень 25(OH)D в сыворотке крови участников составил 20,87 нг/мл (табл. 2). При анализе первичной конечной точки в популяциях по географическим регионам было выявлено, что процент субъектов с дефицитом витамина D колеблется от 29,55% (Владивосток) до 82,22% (Кызыл), с недостаточностью и дефицитом витамина D — от 63,83% (Тюмень) до 93,48% (Западное Заполярье). Результаты оценки первичной точки в зависимости от географического региона приведены в таблице 3.

**Table:** Таблица 2. Средний уровень 25(OH)D (нг/мл) и распространенность дефицита и недостаточности 25(OH)D, %

Географический регион	Распространенность дефицита и недостаточности 25(OH)D, %	Средний уровень 25(OH)D, нг/мл
Владивосток	75	26,77
Екатеринбург	85,71	20,95
Западное Заполярье	93,48	19,96
Кызыл	91,11	14,73
Москва	86,67	18,69
Новосибирск	81,82	22,27
Норильск	81,4	23,42
Ростов-на-Дону	92,86	16,21
Санкт-Петербург	91,49	19,8
Тюмень	63,83	25,74
Всего в исследовании	84,27	20,87

 

**Table:** Таблица 3. Сводная таблица результатов исследования: уровень общего 25(OH)D в сыворотке крови, распределение по географическим регионам

I период исследования
Уровень общего 25(ОН)D в сыворотке крови	Географический регион
Владивосток	Екатеринбург	Западное Заполярье	Кызыл	Москва	Новосибирск	Норильск	Ростов-на-Дону	Санкт-Петербург	Тюмень	Всего
Дефицит, %	29,55	61,90	50,00	82,22	60,00	54,55	51,16	73,81	61,70	40,43	56,40
Недостаточность, %	45,45	23,81	43,48	8,89	26,67	27,27	30,23	19,05	29,79	23,40	27,87
Дефицит или недостаточность суммарно, %	75,00	85,71	93,48	91,11	86,67	81,82	81,40	92,86	91,49	63,83	84,27
Норма, %	25,00	14,29	6,52	8,89	13,33	18,18	18,60	7,14	8,51	36,17	15,73

Демографические показатели. Следующим этапом настоящего исследования явился анализ демографического статуса обследуемых лиц. В исследовании наблюдалась выраженная взаимосвязь низкого уровня витамина D с возрастом участников (p=0,015, сравнение групп с помощью непараметрического критерия Манна–Уитни). Наиболее выраженный дефицит витамина D установлен у молодых людей в возрастной подгруппе 18–25 лет (72,22%) (рис. 1). В общем недостаточность и дефицит витамина D выявлены у 89,81% субъектов в данной возрастной подгруппе. С учетом статистического «взвешивания» в целом по РФ 89,92% молодых людей в возрасте 18–25 лет испытывают недостаточность или дефицит витамина D. При оценке половых особенностей отмечается статистически достоверное преобладание дефицита и недостаточности 25(ОН)D у лиц мужского пола по сравнению с женщинами (p=0,021, критерий χ2 Пирсона) (табл. 5, рис. 2). Поскольку наблюдаемое распределение участников исследования по половому признаку (31:69) отличается от полового распределения населения РФ в анализируемых географических регионах, дополнительно в таблице 4 приведены результаты основного анализа исследования (оценки пропорции участников с дефицитом и недостаточностью витамина D) с учетом статистического «взвешивания» данных. Таким образом, с учетом «взвешивания» данных, в 55,96% случаев наблюдается дефицит витамина D, а в 84,01% — уровень его дефицита или недостаточности. По результатам корреляционного анализа можно сделать вывод о статистически значимой корреляции возраста, пола и расы участников исследования с 25(OH)D в сыворотке крови. В таблице 6 отмечена положительная корреляция уровня 25(ОH)D c возрастом (чем старше участник, тем выше концентрация); а также имеющие место половые и расовые различия относительно уровня 25(OH)D (у лиц женского пола концентрации в среднем выше, чем у лиц мужского пола; у лиц европеоидной расы концентрации выше, чем у лиц монголоидной расы).

**Figure:**
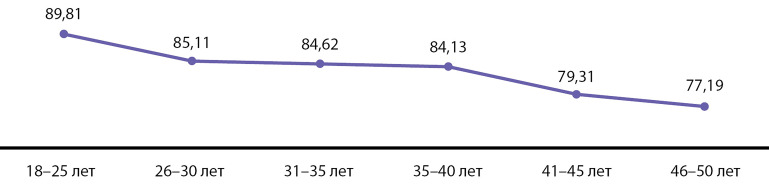
Рисунок 1. Динамика показателей дефицита и недостаточности витамина D в зависимости от возраста, % (n= 445)

**Figure:**
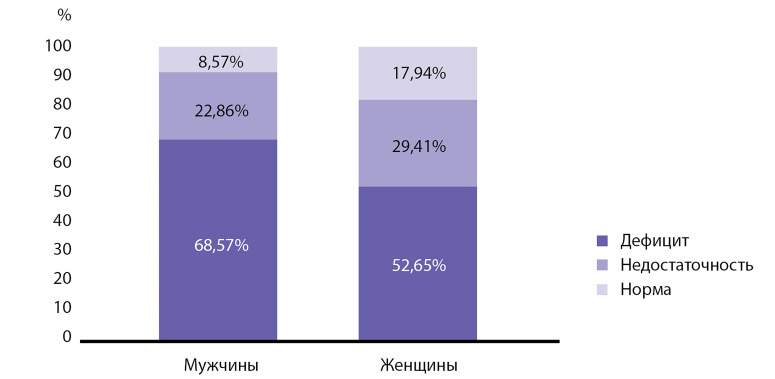
Рисунок 2. Уровень обеспеченности витамином D в зависимости от пола (n=445).

 

**Table:** Таблица 4. Сводная таблица результатов исследования после «взвешивания» данных: уровень общего 25(OH)D в сыворотке крови, распределение по географическим регионам

I период исследования, уровень 25(ОН)D в сыворотке крови
Географический регион	Владивосток	Екатеринбург	Западное Заполярье	Кызыл	Москва	Новосибирск	Норильск	Ростов-на-Дону	Санкт-Петербург	Тюмень	По РФ в целом
Соотношение м:ж	48:52	46:54	47:53	48:52	46:54	47:53	47:53	46:54	47:53	48:52	46:54
Дефицит, %	30,00	61,47	50,00	82,14	58,92	53,83	50,52	73,05	61,20	40,10	55,96
Недостаточность, %	45,00	23,67	43,31	8,76	27,39	27,70	30,49	19,60	30,37	23,49	28,05
Недостаточность или дефицит суммарно, %	75,00	85,14	93,31	90,89	86,31	81,53	81,01	92,65	91,57	63,59	84,01
Норма, %	25,00	14,86	6,69	9,11	13,69	18,47	18,99	7,35	8,43	36,41	15,99

 

**Table:** Таблица 5. Корреляция демографических показателей с уровнем 25(OH)D

Пол	Наличие низкого уровня витамина D	Отсутствие низкого уровня витамина D	В целом по исследованию
Женский	Абс.	279	61	340
%	74,40%	87,14%	76,40%
Мужской	Абс.	96	9	105
%	25,60%	12,86%	23,60%
Различия между группами	Критерий χ2 Пирсона	0,021

 

**Table:** Таблица 6. Корреляция антропометрических показателей с уровнем 25(OH)D

Показатели	N	Spearman R	t(N-2)	p	Наличие корреляции
Возраст	445	0,224	4,842	0,000	да
Пол	445	0,161	3,429	0,001	да
Раса	436	-0,195	-4,148	0,000	да

Нежелательные явления

В ходе исследования нежелательные явления зарегистрированы не были.

## ОБСУЖДЕНИЕ

Резюме основного результата исследования

В представленном исследовании впервые дана комплексная оценка частоты дефицита и недостаточности витамина D среди жителей, проживающих в различных географических широтах страны.

Обсуждение основного результата исследования

Результаты различных эпидемиологических исследований, в которых проводились измерения уровней 25(OH)D в сыворотке крови, позволяют утверждать, что в настоящее время не менее 50% населения Земли имеют низкую обеспеченность витамином D [19–22]. В зону риска дефицита и недостаточности витамина D попадают все жители, проживающие севернее 35-й параллели, что соотносится со всей территорией РФ, Казахстана, Монголии, Турции, Европы, а также практически всей Северной Америки и Китая. Регионы, располагающиеся на данной географической широте, получают недостаточно УФ-излучения, в особенности в осенне-зимние месяцы, что делает синтез витамина D из солнечного света практически невозможным [23]. Кроме этого, многочисленные экологические факторы, в особенности постоянно нарастающий уровень загрязненности атмосферы за счет пыли и промышленных отходов, могут ослабить воздействие УФ-излучения спектра В и тем самым препятствовать адекватной выработке витамина D [24, 25]. Крайняя северная (окрестности мыса Флигели, Земля Франца-Иосифа, Архангельская область 81° с.ш.), южная (непоименованная на картах точка с высотой свыше 3500 м расположена в 2,2 км к востоку от горы Рагдан и к юго-западу от гор Несен (3,7 км) и Базардюзю (7,3 км), Дагестан 41°с.ш.), восточная (остров Ратманова, Чукотский автономный округ 65°с.ш.) и западная (погранзастава Нормельн, Балтийская коса, Калининградская область 54°с. ш.) географические точки на карте России попадают в пояс территорий, дефицитных по витамину D. В связи с этим в РФ проведен ряд исследований, результаты которых согласуются с мировыми данными и подтверждают повсеместную распространенность низких уровней витамина D среди всех возрастных групп населения страны [18]. Однако полученные данные об эпидемиологическом статусе дефицита и недостаточности витамина D в популяции жителей России нельзя считать однозначными ввиду разнообразной исследуемой популяции по половым и возрастным характеристикам, по хроническим заболеваниям и факторам риска низкого статуса витамина D, а также сезона проведения исследования. К тому же отсутствие в проведенных исследованиях единого протокола, критериев включения/исключения, а также стандартизированной методики лабораторной диагностики вносит дискоординацию в интерпретацию и унификацию полученных результатов. Это в совокупности и определило актуальность проведения обширного эпидемиологического исследования, охватывающего регионы, которые представляют собой репрезентативную, с географической точки зрения, выборку регионов РФ с высоким риском развития недостаточного уровня витамина D.

Основным критерием оценки первичной конечной точки проведенного исследования явилось определение процента участников с низким уровнем витамина D, выявленное путем измерения уровня 25(ОН)D в сыворотке крови. В проведенном исследовании средний уровень 25(OH)D в крови участников исследования составил 20,87 нг/мл, что соответствует уровню недостаточности витамина D, при этом находится близко к границе с D-дефицитом. При этом в половине исследуемых территорий (Москва, Санкт-Петербург, Ростов-на-Дону, Западное Заполярье, Кызыл) уровень 25(OH)D соответствовал значениям ниже 20 нг/мл, что свидетельствует в пользу достаточно широкой распространенности его дефицита. Также стоит отметить регионы с наименьшим значением низких показателей 25(OH)D — это города Тюмень (25,74 нг/мл) и Владивосток (26,77 нг/мл). Полученные данные соотносятся и с данными общей распространенности дефицита и недостаточности витамина D и находятся в диапазоне 63,83–93,48%, где наибольшие показатели зарегистрированы в Западном Заполярье (93,48%) и Ростове-на-Дону (92,86%), а наименьшие — в Тюмени (63,83%) и Владивостоке (75%). Попытки оценить уровень распространенности дефицита и недостаточности витамина D уже предпринимались в различных популяциях отдельно взятых регионов страны. Так, например, среди посетителей поликлиники московского региона оценка проведена в работе, в которую были включены 565 пациентов (373 женщины, 192 мужчины). По ее результатам, оптимальная концентрация витамина D выявлена у 38% пациентов (n=213); (39,7±9,6 нг/мл), дефицит витамина D — у 25% (n=141); (16,2±3,4 нг/мл), а недостаточность — у 37% (n=211); (24,9±2,5 нг/мл). Таким образом, у 62% пациентов отмечается низкий уровень витамина D [26], что на 24,67% ниже в сравнении с данными, полученными по Москве в настоящем исследовании (86,67%). При анализе опубликованного материала исследователей из северо-западного региона РФ, где уровень обеспеченности витамином D изучался среди 1569 жителей г. Санкт-Петербурга и г. Петрозаводска, было отмечено, что показатели недостаточности и дефицита витамина D встречаются чрезвычайно часто — в 83,2% [27] случаев, что практически сопоставимо с проведенным исследованием, где эта цифра достигла 91,49%. В то же время изучение содержания 25(ОН)D в сыворотке крови у 5335 пациентов г. Ростова-на-Дону показало, что дефицит витамина D был установлен у 2314 (43,3%), уровень недостаточности 25(ОН)D — у 2066 (38,8%), а оптимальные значения — у 943 (17,6%) пациентов. Суммарно неадекватно низкий статус витамин D относительно нормативных значений выявлен у 82,1% обследованных [28], тогда как в настоящем исследовании — 92,86%. Также высокая частота низкого уровня витамина D подтверждена среди взрослого населения, проживающего на территории Тюменского региона (n=440). По результатам недостаточность зарегистрирована в 22,0% случаев, а дефицит — у 70,7% [29] в сравнении с 63,83% в настоящем исследовании.

Базовые демографические характеристики, такие как пол, возраст и раса, были взяты за основу для оценки вторичной конечной точки. В проведенном исследовании отмечена большая частота дефицита витамина D у лиц мужского пола, а также в подгруппе молодых людей от 18 до 25 лет. Стоит отметить, что полученные результаты не соотносятся с данными опубликованной литературы [8][29], в которых отсутствуют статистически достоверные данные о взаимосвязи 25(OH)D с половыми или возрастными характеристиками обследуемых лиц. Тем не менее полученная информация может свидетельствовать «о скрытом алиментарном голоде относительно витамина D» ввиду измененного высококалорийного, но дефицитного по микронутриентному составу характера питания. Полученную корреляцию уровня 25(OH)D с монголоидной расой можно охарактеризовать в рамках смуглого цвета кожи, при которой может потребоваться в 3–5 раз более длительное пребывание под солнечными лучами для выработки аналогичного количества витамина D в сравнении со светлокожими людьми [30]. К тому же антропометрические особенности, образ жизни, привычки питания, клиническая симптоматика, потребление медицинских ресурсов (количество дней нетрудоспособности, количество посещений врачей общей практики и специалистов в год, количество госпитализаций), а также прием медикаментозных препаратов и наличие определенных хронических заболеваний рассматриваются как значимые факторы риска развития D-дефицита, что требует дополнительного анализа и оценки в исследуемой популяции. В совокупности все эти данные и определяют перспективность дальнейшего изучения вопросов распространенности дефицита и недостаточности витамина D в РФ.

Резюмируя совокупность полученных результатов и опубликованных литературных данных, проблему дефицита витамина D можно классифицировать как пандемию, характерную для всех регионов страны. Именно поэтому изучение распространенности обеспеченности витамином D не теряет своей актуальности и приобретает первостепенное значение для решения вопросов своевременной профилактики, ранней диагностики и адекватного лечения низкого статуса витамина D, которые определены как ключевые причины, необходимые для укрепления здоровья и улучшения качества жизни населения [31].

Направление дальнейших исследований

Дальнейшее изучение проблемы дефицита и недостаточности витамина D в России заключается в проведении второй осенне-зимней волны для анализа совокупности полученных результатов в течение всего календарного года, а также оценке факторов риска развития низкого статуса витамина D.

## ЗАКЛЮЧЕНИЕ

Начало XXI в. можно ознаменовать второй эпохой дефицита витамина D, после победы над рахитом в середине XX в. Высокая распространенность низкой обеспеченности витамином D на территории РФ обосновывает необходимость изучения факторов риска развития D-дефицитных состояний, а также диктует важность дальнейшего изучения эпидемиологического статуса. Для коррекции дефицита витамина D существует довольно широкий выбор препаратов, содержащих колекальциферол, но большинство из зарегистрированных на отечественном рынке — биологически активные добавки, тогда как только лекарственное средство имеет зарегистрированные показания «лечение недостаточности и дефицита витамина D». Поскольку витамин D относится к жирорастворимым витаминам, основной механизм его всасывания в желудочно-кишечном тракте, как и других жирорастворимых витаминов, — это мицеллирование. Использование препарата, созданного на основе мицеллированного раствора колекальциферола (Аквадетрим®), обусловливает хорошую степень всасывания независимо от состава пищи, приема лекарств или состояния желудочно-кишечного тракта. Аквадетрим® в виде растворимых таблеток — удобная форма витамина D, которую можно растворить как во рту, так и в небольшом количестве воды. Расширение географии дефицита витамина D в РФ в совокупности будет способствовать созданию и развитию образовательных и профилактических программ, что может найти отражение в Национальных клинических рекомендациях.

## References

[cit1] Gaksch Martin, Jorde Rolf, Grimnes Guri, Joakimsen Ragnar, Schirmer Henrik, Wilsgaard Tom, Mathiesen Ellisiv B., Njølstad Inger, Løchen Maja-Lisa, März Winfried, Kleber Marcus E., Tomaschitz Andreas, Grübler Martin, Eiriksdottir Gudny, Gudmundsson Elias F., Harris Tamara B., Cotch Mary F., Aspelund Thor, Gudnason Vilmundur, Rutters Femke, Beulens Joline W. J., van ‘t Riet Esther, Nijpels Giel, Dekker Jacqueline M., Grove-Laugesen Diana, Rejnmark Lars, Busch Markus A., Mensink Gert B. M., Scheidt-Nave Christa, Thamm Michael, Swart Karin M. A., Brouwer Ingeborg A., Lips Paul, van Schoor Natasja M., Sempos Christopher T., Durazo-Arvizu Ramón A., Škrabáková Zuzana, Dowling Kirsten G., Cashman Kevin D., Kiely Mairead, Pilz Stefan (2017). Vitamin D and mortality: Individual participant data meta-analysis of standardized 25-hydroxyvitamin D in 26916 individuals from a European consortium. PLOS ONE.

[cit2] Tagliabue Elena, Raimondi Sara, Gandini Sara (2015). Vitamin D, Cancer Risk, and Mortality. Advances in Food and Nutrition Research.

[cit3] Al Mheid I, Quyyumi AA. Vitamin D and cardiovascular disease: Controversy unresolved. J. Am. Coll. Cardiol. 2017;70:89–100. 28662812 10.1016/j.jacc.2017.05.031

[cit4] Berridge Michael J. (2017). Vitamin D deficiency and diabetes. Biochemical Journal.

[cit5] Altieri Barbara, Muscogiuri Giovanna, Barrea Luigi, Mathieu Chantal, Vallone Carla V., Mascitelli Luca, Bizzaro Giorgia, Altieri Vincenzo M., Tirabassi Giacomo, Balercia Giancarlo, Savastano Silvia, Bizzaro Nicola, Ronchi Cristina L., Colao Annamaria, Pontecorvi Alfredo, Della Casa Silvia (2017). Does vitamin D play a role in autoimmune endocrine disorders? A proof of concept. Reviews in Endocrine and Metabolic Disorders.

[cit6] Fung June L., Hartman Terryl J., Schleicher Rosemary L., Goldman Marlene B. (2017). Association of vitamin D intake and serum levels with fertility: results from the Lifestyle and Fertility Study. Fertility and Sterility.

[cit7] Holick Michael F, Chen Tai C (2018). Vitamin D deficiency: a worldwide problem with health consequences. The American Journal of Clinical Nutrition.

[cit8] Klinicheskie rekomendatsii. Defitsit vitamina D u vzroslykh: diagnostika, lechenie i profilaktika. Obshchestvennaya organizatsiya «Rossiiskaya assotsiatsiya endokrinologov». M.; 2015.

[cit9] EN Grineva, T Karonova, E Micheeva, O Belyaeva, IL Belyaeva (2016). Vitamin D deficiency is a risk factor for obesity and diabetes type 2 in women at late reproductive age. Aging.

[cit10] Markova T.N., Markov D.S., Markelova T.N., i dr. Rasprostranennost' defitsita vitamina D i faktorov riska osteoporoza u lits molodogo vozrasta // Vestnik Chuvashskogo Universiteta. — 2012. — T. 234. — №3. — S. 441-446.

[cit11] Filatova T.E., Davydov V.V., Nizov A.A. i dr. Obespechennost' vitaminom D patsientov s sakharnym diabetom 2 tipa i izbytochnym vesom¸ prozhivayushchikh v Ryazanskoi oblasti/Sakharnyi diabet — pandemiya XXI. Sbornik tezisov VIII (XXV) Vserossiiskogo diabetologicheskii kongress s mezhdunarodnym uchastiem. FGBU «NMITs endokrinologii» Minzdrava Rossii; OOO «Rossiiskaya assotsiatsiya endokrinologov». 2018. 100 s.

[cit12] Романцова, Romantsova Elena, Борисенко, Borisenko Elena, Бабцева, Babtseva Albina (2016). VITAMIN D PROVISION FOR CHILDREN AND ADULT POPULATION OF THE AMUR REGION. Bulletin physiology and pathology of respiration.

[cit13] Khazova E.L., Shirinyan L.V., Zazerskaya I.E., i dr. Sezonnye kolebaniya urovnya 25-gidroksikholekal'tsiferola u beremennykh, prozhivayushchikh v Sankt-Peterburge // Ginekologiya. — 2015. — T. 17. — № 4. — S. 38-42.

[cit14] Malyavskaya S.I., Kostrova G.N., Lebedev A.V. Urovni vitamina D u predstavitelei razlichnykh grupp naseleniya goroda Arkhangel'ska // Ekologiya cheloveka. — 2018. — T. 356. — №1. — S. 60-64.

[cit15] Nurlygayanov R.Z., Syrtlanova E.R. Rasprostranennost' defitsita vitamina D u lits starshe 50 let, postoyanno prozhivayushchikh v respublike Bashkortostan, v period minimal'noi insolyatsii // Osteoporoz i osteopatii. — 2012. — T. 15. — № 3. — S. 7-9.

[cit16] Nurlygayanov R Z, Syrtlanov E R, Minasov T B, Borisov I V (2018). THE LEVEL OF VITAMIN D IN PEOPLE OLDER THAN 50 YEARS RESIDING IN THE REPUBLIC OF BASHKORTOSTAN IN THE PERIOD OF MAXIMUM INSOLATION. Osteoporosis and Bone Diseases.

[cit17] Spasich T.A., Lemeshevskaya E.P., Reshetnik L.A., i dr. Gigienicheskoe znachenie defitsita vitamina D u naseleniya Irkutskoi oblasti i puti ego profilaktiki // Acta Biomedica Scientifica. — 2014. — T. 100. — № 6. — S. 44–47.

[cit18] Petrushkina Alexandra A., Pigarova Ekaterina A., Rozhinskaya Liudmila Y. (2019). The prevalence of vitamin D deficiency in Russian Federation. Osteoporosis and Bone Diseases.

[cit19] Lee Joyce M., Smith Jessica R., Philipp Barbara L., Chen Tai C., Mathieu Jeffrey, Holick Michael F. (2007). Vitamin D Deficiency in a Healthy Group of Mothers and Newborn Infants. Clinical Pediatrics.

[cit20] Holick Michael F. (2008). Vitamin D Status: Measurement, Interpretation, and Clinical Application. Annals of Epidemiology.

[cit21] Kumar Juhi, Muntner Paul, Kaskel Frederick J., Hailpern Susan M., Melamed Michal L. (2009). Prevalence and Associations of 25-Hydroxyvitamin D Deficiency in US Children: NHANES 2001–2004. Pediatrics.

[cit22] Béghin Laurent, Huybrechts Inge, Vicente-Rodríguez German, De Henauw Stefaan, Gottrand Frédéric, Gonzales-Gross Marcela, Dallongeville Jean, Sjöström Michael, Leclercq Catherine, Dietrich Sabine, Castillo Manuel, Plada Maria, Molnar Dénes, Kersting Mathilde, Gilbert Chantal C, Moreno Luis A (2012). Main characteristics and participation rate of European adolescents included in the HELENA study. Archives of Public Health.

[cit23] Kimlin Michael G., Olds William J., Moore Michael R. (2006). Location and Vitamin D synthesis: Is the hypothesis validated by geophysical data?. Journal of Photochemistry and Photobiology B: Biology.

[cit24] Parisi Alfio V., Turnbull David J., Downs Nathan J. (2012). Influence of high levels of cloud cover on vitamin D effective and erythemal solar UV irradiances. Photochemical & Photobiological Sciences.

[cit25] Kimlin Michael G. (2008). Geographic location and vitamin D synthesis. Molecular Aspects of Medicine.

[cit26] Poluektova A.Yu., Martynova E.Yu., Fatkhutdinov I.R., Demidova T.Yu., Poteshkin Yu.E. (2018). Genetic features of sensitivity to vitamin D and prevalence of vitamin D deficiency among outpatients. Russian Journal of Woman and Child Health.

[cit27] AGUREEVA O V, ZhABREVA TO, SKVORTsOVA E A, LUGOVSKAYa G I, SYChIK E V (2017). ANALIZ UROVNYa VITAMINA D V SYVOROTKE KROVI PATsIENTOV V ROSTOVSKOY OBLASTI. Osteoporosis and Bone Diseases.

[cit28] Karonova T.L., Grineva E.N., Nikitina I.L., i dr. Uroven' obespechennosti vitaminom D zhitelei Severo-Zapadnogo regiona RF (g. Sankt-Peterburg i g. Petrozavodsk) // Osteoporoz i osteopatii. — 2013. — T. 16. — № 3. — S. 3–7.

[cit29] Suplotova Liudmila A., Avdeeva Valeria A., Rozhinskaya Liudmila Y. (2019). Vitamin D status in residents of Tyumen region. Obesity and metabolism.

[cit30] Clemens T.L., Henderson S.L., Adams J.S., Holick M.F. (2003). INCREASED SKIN PIGMENT REDUCES THE CAPACITY OF SKIN TO SYNTHESISE VITAMIN D3. The Lancet.

[cit31] Bjelakovic Goran, Gluud Lise Lotte, Nikolova Dimitrinka, Whitfield Kate, Wetterslev Jørn, Simonetti Rosa G, Bjelakovic Marija, Gluud Christian (2014). Vitamin D supplementation for prevention of mortality in adults. Cochrane Database of Systematic Reviews.

